# Qualitative analysis of out-of-hospital self-management capabilities and ongoing care needs in patients with gynecological malignancies and venous thromboembolism

**DOI:** 10.1080/07853890.2026.2628359

**Published:** 2026-02-14

**Authors:** Tian-Hua Li, Yan-Wei Wang, Jing-Xuan Feng, Ning Sun, Ya-Jing Bian, Jun-Ying Ma, Lai-You Li

**Affiliations:** ^a^ Department of Gynecology, Fourth Hospital of Hebei Medical University, Shijiazhuang, Hebei Province, China; ^b^ Department of Clinical Laboratory, Fourth Hospital of Hebei Medical University, Shijiazhuang, Hebei Province, China; ^c^ Department of Nursing, Fourth Hospital of Hebei Medical University, Shijiazhuang, Hebei Province, China

**Keywords:** Continuous care, gynecological malignancies, qualitative research, self-management ability, venous thromboembolism

## Abstract

**Background:**

Postoperative patients with gynecological malignant tumors are prone to venous thromboembolism (VTE) due to hypercoagulability and limited mobility, and lack of professional guidance after discharge, further increasing the risk of VTE occurrence. Therefore, this study focuses on the current status of self-management ability and continuity of care needs of patients after discharge, aiming to clarify the practical difficulties of prevention activities such as limb activity and symptom recognition in the home environment, in order to improve the implementation and compliance of VTE prevention measures for patients outside the hospital.

**Methods:**

Utilizing a phenomenological approach within qualitative research, interview outlines were developed through a comprehensive literature review and expert consultations. The interviewees were selected from 10 patients with gynecological malignant tumors who underwent chemotherapy in our department within 3 months after surgery. The interview method is semi-structured and in-depth qualitative interviews. Data were transcribed, coded, refined, and analyzed utilizing the Colaizzi phenomenological 7-step analysis method.

**Results:**

The self-management abilities for out-of-hospital VTE prevention among patients with gynecological malignancies were categorized into five themes: inadequate VTE risk perception, low self-efficacy in VTE prevention, limited interest in VTE-related information, insufficient VTE prevention education and awareness efforts by healthcare providers, and restricted access to VTE prevention resources. Ongoing care needs were identified into two primary areas: a desire for diverse VTE prevention educational materials and support from families and relatives.

**Conclusion:**

Significant challenges persist in the out-of-hospital self-management and ongoing care of VTE among patients with gynecological malignancies. To address these issues, healthcare providers must develop effective strategies to enhance self-management, optimize continuous care services, and provide comprehensive information resources and social support. These interventions aim to improve patient adherence to VTE prevention measures and enhance their efficacy.

## Introduction

Venous thromboembolism (VTE), encompassing deep vein thrombosis of the lower extremities and pulmonary embolism, arises from abnormal blood coagulation and subsequent occlusion of blood vessels within the venous system. It is a common complication in cancer patients. Notably, the incidence of postoperative VTE in patients with gynecological malignancies can reach as high as 5.9%, significantly impacting their quality of life and long-term prognosis [1–3]. As advancements in fast-track recovery protocols shorten postoperative hospital stays, the risk of VTE extends beyond the inpatient setting. Implementing effective prevention strategies for high-risk patients has the potential to reduce VTE incidence from 30% to 80% [4].

However, suboptimal patient compliance and low implementation rates remain significant challenges in an out-of-hospital setting [5].

The prevention of VTE outside the hospital, which is the focus of this study, refers to the process of reducing the risk of VTE through a series of planned intervention measures after patients are discharged from direct medical monitoring in the hospital. Specifically, this includes continuous lower limb functional exercise, active management of water and activity levels, early identification of abnormal symptoms, and timely medical treatment. In this study, ‘self-management ability’ specifically refers to the ability of patients to independently or with the assistance of family members complete VTE prevention related tasks in their home environment after discharge, covering three dimensions: first, cognitive level, that is, whether patients understand the harm, prevention measures, and importance of VTE; The second aspect is the skill level, which refers to the ability to correctly perform specific behaviors such as lower limb activities; The third is at the regulatory level, which means being able to cope with difficulties such as limited mobility and maintain long-term compliance. The definition of this concept is based on Orem’s self-care theory, emphasizing the ability of individuals to meet their health needs through self-care in a diseased state, as well as the process of improving self-efficacy through learning and adaptation.

The research site is the gynecology department of a tertiary hospital in Hebei Province. The hospital has established standardized risk assessment and in-hospital prevention procedures for VTE in the management of gynecological malignant tumors during the perioperative period. However, the continuity of prevention after discharge still relies on the autonomy of patients and their families, and there are problems such as unsystematic education content and insufficient continuity support. Therefore, this study intends to adopt a qualitative research method and conduct semi-structured interviews to gain a deeper understanding of patients’ real experiences, obstacles, and needs for continuity of care during VTE prevention outside the hospital. The specific connotation and practical difficulties of self-management will be clarified, providing a scientific basis for constructing personalized continuity of care plans centered on patients.

## Methods

### Research participants

This study used purposive sampling method. In order to increase patients’ personal experience and make them more aware of the problems encountered in the self-management process of VTE outside the hospital, this study selected patients who were readmitted for chemotherapy within 3 months after gynecological malignant tumor surgery in a tertiary hospital in Hebei Province from January to June 2024 for in-depth semi-structured interviews. The inclusion criteria were as follows: (1) Postoperative patients with gynecological tumors; (2) Patients whose condition does not worsen within 3 months after surgery but requires readmission for chemotherapy based on pathological type; (3) VTE high-risk patients (patients with Caprini score ≥ 5); (4) voluntary participation and provision of informed consent.

The exclusion criteria encompassed patients with (1) end-stage diseases; (2) lower extremity mobility impairment; (3) lower extremity skin lesions or inflammation; and (4) a history of mental illness.

The sample size for the interviews was determined through data saturation, and this continued until new themes emerged. A total of 10 female patients with gynecological malignancies (coded A01 to A10) were enrolled in the study. Participant demographics are provided in Table 1. This study received approval from the Hospital Ethics Committee (Ethics number: 2023KY133).

**Table 1.** General information of respondents.

Number	Age (years)	Education level	Occupation	Place of residence	Disease diagnosis	Residential status	VTE history
A01	59	High school	Staff	City	Ovarian cancer (Stage: IVA)	Living alone	None
A02	62	High school	Staff	City	Ovarian cancer (Stage: IVA)	Living with children	None
A03	69	Primary school	Unemployed	Countryside	Ovarian cancer (Stage: IIIC)	Living alone	None
A04	54	Primary school	Unemployed	Countryside	Ovarian cancer (Stage: IVA)	Living alone	None
A05	73	Middle school	Farmer	Countryside	Ovarian cancer (Stage: IIIC)	Living with children	None
A06	61	Middle school	Unemployed	Countryside	Endometrial cancer	Living alone	None
A07	59	High school	Laborer	Town	Endometrial cancer	Living alone	None
A08	45	Junior college	Staff	Town	Ovarian cancer (Stage: IIIC)	Living alone	None
A09	52	Middle school	Laborer	Town	Endometrial cancer	Living with children	None
A10	61	Primary school	Laborer	Town	Endometrial cancer	Living with children	None

## Research methods

### Design an interview outline

Based on the research objectives and content, the interview outline was developed through literature review, research group discussions, and interview planning. After conducting pre interviews with two interviewees, the interview outline was adjusted and improved to ultimately form a formal interview outline. The final interview content comprised the following questions: (1) What is your understanding of venous thrombosis and its associated risks? (2) Are you aware of the signs or symptoms indicative of venous thrombosis? (3) What are the risk factors for venous thrombosis according to your understanding? (4) What methods are available for preventing venous thrombosis and how did you acquire this information? (5) Which prevention method do you prefer and are willing to consistently adhere to? (6) How do you perceive out-of-hospital venous thrombosis prevention? Do you consider it necessary? Why or why not? (7) What actions did you undertake following hospital discharge? (8) What factors contributed to any non-adherence or reluctance to follow recommended prevention measures? Would your health behaviors change if these barriers were addressed? (9) What difficulties or challenges have you encountered in managing venous thrombosis prevention out-of-hospital? (10) What are your specific needs regarding venous thrombosis prevention after hospital discharge? (11) Have you actively sought information about thrombosis prevention? If so, how did you obtain this information? (12) What type of assistance would you find helpful from your healthcare provider and in what format would you prefer to receive it?

### Data collection

This is a phenomenological study using a semi-structured in-depth interview method to collect data, conducting face-to-face in-depth interviews with the included research subjects according to the interview outline. The interviewer is an independent nursing professional who has not participated in previous intervention measures and has undergone systematic qualitative research and learning. The interview location should be a demonstration room with good sound insulation and no external environmental interference in the ward. The interview site should avoid the presence of unrelated personnel (other patients or family members outside the research team) to prevent information leakage.

Before the interview, explain to the interviewee the purpose, method, importance of on-site recording and transcript, promise to anonymize all information, encrypt and store the recording files, and limit access to the research team only. At the same time, emphasize to the interviewee that they have the right to refuse to participate in the interview, and express understanding and respect when the interviewee refuses. Obtain the interviewee’s consent and sign the informed consent form before starting the interview.

The entire interview process creates a relaxed and pleasant atmosphere, paying attention to observing and recording nonverbal information such as the interviewee’s facial expressions and movements, and using interview techniques such as rhetorical questions, questioning, repetition, summarization, and response. Avoid any suggestive behavior that may affect the interviewee and try to understand their true feelings and behavior. The researcher himself always maintained an objective perspective and a neutral attitude during the research process. The interview will last for about 20 min, and at the end of the interview, ask the interviewee if there is anything else they want to express to ensure that their viewpoint is fully expressed.

### Data organization and analysis

Within 24 h after the interview, the researchers used software to transcribe the entire recording into a verbatim transcript. During the transcription process, attention was paid to the position of punctuation marks and the correct interweaving of the interviewee’s voice, pauses, facial expressions, and body movements. Inconsistent, confusing, or easily revealing oral language was cleverly transformed into correct and fluent written language, enhancing document readability. Finally, the transcribed interview transcript will be returned to the interviewee for verification, and any inaccurate statements will be corrected to ensure the reliability of the data.

The Colaizzi 7-step analysis method was used to analyze the data, and two researchers independently manually coded them [6]: (1) immersion in the data through repeated listening and reading of transcripts. (2) analyzing the text word by word and line by line, using an iterative recursive analytic process to accurately interpreting segments to identify significant statements; (3) organization of recurring and meaningful ideas into coding table; (4) development of preliminary themes through data classification and summarization; (5) comprehensive theme description ensuring no details are omitted; (6) theme refinement through comparative analysis and induction of coded data; (7) validation of findings by participant to ensure accuracy and authenticity. If new text is found to fully conform to the existing framework during this process, it indicates saturation; If new concepts emerge, it is necessary to return to stage one, modify the coding, and update the framework.

During the process of topic extraction, if there are any objections to the encoding of the two, we will review the context of the questionable paragraphs together in the original interview data. Through repeated discussions, we will reach a consensus on the essence of the statement. If we cannot reach a consensus after discussion, a third senior researcher will be introduced as an ‘arbitrator’ to ensure that the encoding is faithful to the interviewee’s original intention.

## Results

We identified five primary themes related to self-management of out-of-hospital VTE prevention among patients with gynecological malignancies: inadequate VTE risk perception, low self-efficacy in VTE prevention, limited interest in VTE-related information, insufficient education and awareness efforts on VTE prevention by healthcare providers, and restricted access to VTE prevention resources.

Furthermore, two primary areas of ongoing care were identified: a need for diverse forms of education and the need for support from family and relatives (Table 2).

**Table 2.** Theme and categories with associated subcategories.

Theme	Category	Example citation
Out-of-hospital self-management ability of VTE in patients with gynecological malignancies	Inadequate VTE risk perception	‘I don’t know what venous thrombosis is’
	Low self-efficacy	‘At that time, my stomach hurt so much so I could not do it’.
	Low demands for VTE-related knowledge	‘Ha-ha, I did not think about it (referring to the demand for VTE prevention). I would probably take medicine if it did not work (referring to that after the occurrence of VTE)’.
	Lack of education and awareness efforts on VTE prevention by healthcare providers	‘Well, no, this does not mean that I have VTE, right?’
	Single access to obtain resources	‘The nurse told me about it before the operation’
Continuous care demands of VTE in patients with gynecological malignancies	Demand for various forms of education and awareness efforts	‘Just send a leaflet, it is more intuitive. If you build a WeChat group, sometimes older patients might not get used to it’.
	Demands for support from families and relatives	‘I cannot read and I cannot use a mobile phone. Just send across a piece of paper, and I will let my child read it to me when it’s possible’.

After analysis, it was found that patients with different social characteristics have certain differences in cognition and needs. Elderly and rural patients have a more obvious lack of understanding of VTE. Compared with patients living alone, patients living with their children have better compliance and efficacy, and patients of different age groups have different needs for educational forms. The specific theme and related case analysis are as follows.

## Out-of-hospital self-management ability of VTE in patients with gynecological malignancies

### Inadequate VTE risk perception

Participants demonstrated a limited understanding of VTE. Only two of the ten interviewees exhibited basic knowledge of the condition, while the remaining eight either confused VTE with cerebral thrombosis or were entirely unfamiliar with the term. For instance: patient A01 stated: ‘I don’t know what venous thrombosis is’, while Patient A05 described it as ‘a blood clot’. Patient A06 inquired ‘Venous thrombosis? Is that cerebral thrombosis?’ Furthermore, most participants displayed a lack of awareness regarding VTE-related symptoms, risk factors, and potential complications. Some confused VTE with neurological symptoms caused by chemotherapy or cerebral thrombosis. For example, Patient A02 expressed uncertainty about the reasons for their increased VTE risk, stating, ‘I don’t know about any of this. The doctor mentioned I am prone to it, but I don’t understand why’. Patient A05 described experiencing a sensation of tightness around their feet and wondered if it could be a blood clot. Patient A08 associated VTE with a sense of weakness, based on their knowledge of cerebral thrombosis.

### Low self-efficacy

Patient self-efficacy, defined as the ability to comprehend and effectively implement VTE prevention measures, was found to be low among patients. This was primarily attributed to two factors: (1) Inability to effectively grasp VTE prevention measures: Many patients struggled to understand and implement VTE prevention measures correctly. For example: Patient A05 described that they performed increased physical activity, including ankle pump exercises, without adhering to standardized guidelines. Patient A06 expressed uncertainty about how to prevent VTE by asking, ‘How can I prevent it?’ (2) Personal physiological and external environmental factors reducing compliance: For example, Patient A01 reported a lack of motivation to perform ankle pump exercises, stating, ‘I did not do it and I do not plan to do it. I do not have motivation and I only did it when I was here for surgery’. Patient A02 reported difficulty in performing ankle pump exercises due to postoperative abdominal pain, stating, ‘At that time, my stomach hurt so much so I could not do it’. Patient A04 expressed discomfort and inconvenience associated with wearing compression stockings, stating, ‘Ah, I do not wear it, it is hard to put on and take it off, and it is troublesome, so I do not wear it’. Patient A09 described removing compression stockings due to heat-related discomfort, explaining, ‘It was really hot then, I really could not wear it, so I took it off’.

### Low demands for VTE-related knowledge

The majority of participants exhibited minimal or no interest in seeking additional VTE prevention knowledge, relying primarily on the limited guidance and education provided by healthcare providers. Their lack of understanding of VTE contributed to their unawareness of the need for VTE prevention. For example: Patient A02 remarked: ‘Ha-ha, I did not think about it (referring to the demand for VTE prevention). I would probably take medicine if it did not work (referring to that after the occurrence of VTE).’ When asked about additional VTE prevention measures post-discharge, Patient A03 admitted, ‘I will do what the doctor tells me to do’. Patient A07 further emphasized this reliance on healthcare professionals by stating, ‘I do not know anything else, I do not know much’.

### Lack of education and awareness efforts on VTE prevention by healthcare providers

During the interview process, most patients had insufficient understanding of VTE prevention education content. Healthcare providers often face time constraints, resulting in limited VTE prevention education for patients. For example: Patient A10 expressed uncertainty regarding the relationship between intermuscular thrombosis and VTE, stating, ‘Well, no, this does not mean that I have VTE, right? (after the occurrence of intermuscular thrombosis). No one knows whether to treat it or not’.

### Single access to obtain resources

The majority of the study participants, being middle-aged and elderly patients, had limited access to information and primarily relied on healthcare providers for guidance. For example, when asked about acquiring VTE knowledge, Patient A05 stated, ‘The nurse told me about it before the operation’. Similarly, Patient A06 confirmed that preoperative nursing education was the sole source of VTE-related information received.

## Continuous care demands of VTE in patients with gynecological malignancies

### Demand for various forms of education and awareness efforts

Participants emphasized the need for tailored VTE prevention education and awareness efforts, highlighting the importance of personalized, and targeted methods. For example, Patient A01 suggested, ‘Just send a leaflet, it is more intuitive. If you build a WeChat group, sometimes older patients might not get used to it’. Patient A03, with limited literacy and technological skills, expressed a preference for paper-based communication, stating, ‘I cannot read or write, and I cannot use a mobile phone. Just send across a piece of paper, and I will let my child read it to me when it’s possible’. In contrast, Patient A06, who had basic digital literacy, appreciated the convenience of electronic platforms, commenting, ‘That is pretty good (referring to the WeChat electronic platform). I am illiterate, but I can still use my mobile phone and watch videos on it’. Patient A07 further emphasized the value of visual aids by suggesting, ‘Anything will do, including the mini videos’.

### Demands for support from families and relatives

Most participants, often due to factors such as illness, limited self-care ability, or advanced age, emphasized the need for family support in managing VTE prevention measures post-discharge. Through interviews, it was found that non solitary patients have higher compliance and stronger implementation of relevant preventive measures. For example, Patient A03, with limited literacy and mobility, highlighted the reliance on family members for information access, stating, ‘I cannot read and I cannot use a mobile phone. Just send across a piece of paper, and I will let my child read it to me when it’s possible’. Patient A06 described difficulties in applying compression stockings and the subsequent reliance on a partner for assistance, stating, ‘My partner helps me wear it. When I first tried it, I did not dare bend down, so I was unable to put it on. It was uncomfortable and tight and it was difficult to pull up’.

## Discussion

This study found that patients with gynecological malignant tumors exhibit a complex characteristic of ‘low self-efficacy high family dependence’ in self-management of VTE outside the hospital, consistent with previous qualitative studies. Patients generally report insufficient knowledge and compliance in VTE prevention, and expect to receive continuous professional guidance and family support after discharge. Compared with similar studies in China, this study further reveals the role of ‘family support’ in continuity of care, providing empirical evidence for incorporating families into the core role of VTE continuity of care [7,8]. Compared with the patient-centered approach in foreign countries, the family oriented approach and the tripartite collaboration of ‘doctor-patient-family’ in the Chinese context are more in line with practical feasibility and cultural expectations, and can serve as the underlying logic for localized intervention design.

### Improve knowledge system of VTE prevention and protection for doctors and patients and self-management efficiency of patients

The findings indicate that patients generally possessed a superficial understanding of VTE prevention and protection, primarily relying on information from healthcare providers. These results align with previous research conducted by Bshabshe et al. and Kim et al. [9,10]. To enhance patient knowledge and engagement in VTE prevention and protection, it is crucial to strengthen their understanding of VTE and increase awareness. A core strategy involves improving healthcare provider education and knowledge. Steps to achieve this include: Establishing a specialized VTE health education professional group, developing a comprehensive VTE educational curriculum, and enhancing the knowledge of healthcare providers through regular specialized training and assessment [11–13]. Furthermore, increasing the frequency of patient education and incorporating regular follow-up will bolster patient awareness and adherence to VTE prevention protocols, ultimately enhancing self-efficacy.

### Optimize health education content and forms of VTE

Research indicates that the optimal form of VTE education utilizes visual stimuli to disseminate information about pathophysiology, prevention, management, and risks in a concise and personalized manner [14]. To this end, we can combine this concept with traditional preaching methods and achieve a shift from ‘knowledge imparting’ to ‘skill empowerment’. In terms of content, obscure medical terms should be abandoned and instead ‘patient language’ and ‘analogical teaching’ should be adopted. Formally, the strategy of ‘visualization + interactivity’ is implemented, using anatomical animations, physical models, and 1-2-minute short videos to assist understanding, and mandatory implementation of the ‘feedback method’ to ensure that patients truly understand and use it. For elderly patients with limited digital literacy, we provide large font manuals, voice broadcasts, and family co managed accounts, supplemented by peer education and narrative medicine, to construct a multidimensional and three-dimensional education matrix, effectively enhancing the self-management ability and risk response awareness of patients and their families.

### Develop continuous care services

Continuous care that bridges inpatient and out-of-hospital settings, provides tailored health guidance and medical support to facilitate post-discharge recovery based on patient-specific nursing needs [15–17]. This care model has gained prominence in China, with nursing professionals increasingly emphasizing post-discharge care. Studies have demonstrated that continuous care significantly enhances treatment compliance, nursing satisfaction, and quality of life in various disease areas, including cardiovascular disease [18], chronic kidney disease [19], and diabetes mellitus [20]. The Ministry of Health has emphasized the importance of ongoing out-of-hospital care and patient follow-up in the *National Nursing Development Program (2021–2025)* [21]. These developments highlight continuous care as a pivotal intervention model for improving patient compliance and outcomes post-discharge. Patient interviews revealed limited access to VTE-related information post-discharge, lack of supportive resources, and significant reliance on healthcare provider guidance. These findings align with research by Pu et al. [22] To address these challenges, healthcare providers should consider the following strategies: establish a VTE continuous care professional group; develop diverse health education platforms tailored to the needs of the patient; utilize these platforms to provide ongoing patient and family support; leverage professional expertise and resources to enhance continuous outpatient care services. Additionally, healthcare providers should encourage active family involvement in the implementation of VTE prevention plans to provide essential support, supervision, comfort, and encouragement to patients.

Compared with the patient-centered approach in foreign countries, the family-oriented approach and the tripartite collaboration of ‘doctor patient family’ in the Chinese context are more in line with practical feasibility and cultural expectations and can serve as the underlying logic for localized intervention design. In the Chinese medical ecosystem, family support is not only an emotional support, but also a safety net for treatment: family members undertake key tasks such as medication reminders, symptom observation, and medical accompaniment. At the same time, patients’ concerns about privacy and sensitive information, unfamiliarity with digital tools, and the urban-rural digital divide can all affect the acceptability and effectiveness of remote follow-up [23]; These findings suggest that promoting digital continuity of care in China requires the principle of ‘low threshold access high trust communication family learning’, while balancing offline accessibility and online humanistic care. In addition, it is also possible to consider incorporating ‘postoperative VTE care for gynecological tumors’ into the hospital’s discharge readiness and quality assessment indicators in the future; Embedding VTE discharge follow-up paths and reminders in electronic medical records; Set up ‘family account co binding’; Promote the synergy between medical consortia and communities.

Although this study delved into the self-management experience and continuity of care needs for VTE in discharged patients with gynecological malignancies, there are still certain limitations. Firstly, the generalizability of the research results is limited. This is a single-center study, and the representativeness of the sample is insufficient. Patients from different regions and levels of medical institutions may have differences in economic level, health literacy, and access to medical resources. Therefore, the research conclusions may not be fully applicable to patient populations in other regions or primary medical institutions. Secondly, recall bias is difficult to avoid. Due to the interview content involving the patient’s long-term home management experience after discharge, some details may have been forgotten or subjectively modified due to the passage of time, which may deviate from objective records. Finally, the subjective influence of individual researchers. Although the Colaizzi phenomenological analysis method was used and double coded to reduce errors, the process of extracting themes and interpreting meanings from interview data was inevitably influenced by the researcher’s personal professional background and subjective experience. Future research may consider conducting longitudinal follow-up surveys with multiple centers and large samples, combined with objective clinical indicators, to further validate and improve relevant conclusions.

## Conclusions

In summary, patients with gynecological malignancies often demonstrate limited VTE- knowledge, limited awareness of personal VTE risks, low self-efficacy, and encounter barriers related to health care providers. Challenges in accessing post-discharge information and support further hinder effective self-management. To address these issues, healthcare providers should implement comprehensive VTE prevention strategies that include: enhancing the content and methods of VTE education, incorporating a range of educational materials and approaches; fostering collaborative partnerships between healthcare providers and patients to standardize the VTE prevention process; strengthening continuous care services to bridge the gap between hospital and home care, ensuring patients receive comprehensive support and information after discharge. By improving educational resources and integrating continuous care, these strategies aim to increase VTE prevention knowledge, patient satisfaction, self-management abilities, compliance, and overall patient outcomes. This approach will help minimize the occurrence of VTE, ultimately improving the quality of life and long-term prognosis of patients. This approach will help improving the quality of life and long-term prognosis of patients.

## Data Availability

The datasets used and/or analyzed during the current study are available from the corresponding author upon reasonable request.
